# Artificial long-day photoperiod enhances growth performance and metabolic indicators in young male goats

**DOI:** 10.1093/jas/skaf270

**Published:** 2025-08-09

**Authors:** Leonardo Iván Vélez, Manuel Jesús Flores, Horacio Hernández, Alexis A Vargas-Cruz, Ricardo Avilés, Cesar A Rosales-Nieto

**Affiliations:** Campo Experimental La Laguna, Instituto Nacional de Investigaciones Forestales, Matamoros, México; Campo Experimental La Laguna, Instituto Nacional de Investigaciones Forestales, Matamoros, México; Centro de Investigación en Reproducción Caprina (CIRCA), Posgrado en Ciencias Agrarias, Universidad Autónoma Agraria Antonio Narro, Torreón, México; Centro de Investigación en Reproducción Caprina (CIRCA), Posgrado en Ciencias Agrarias, Universidad Autónoma Agraria Antonio Narro, Torreón, México; Campo Experimental Las Huastecas, Instituto Nacional de Investigaciones Forestales, Agrícolas y Pecuarias, Altamira, México; Department of Agricultural Sciences, Texas State University, San Marcos, TX, USA

**Keywords:** carcass weight, feed intake, IGF-1, male goats, *Capra hircus*

## Abstract

This study aimed to evaluate whether exposure to artificial long-day photoperiods stimulates growth performance in young male goats, either intact or castrated. Forty young male goats (13 wk old), either intact (**I**) or castrated (**C**), were assigned to one of two photoperiod treatments: artificial photoperiod (**AP**; 16h light:8h dark) or natural photoperiod (**NP**; 12h light:12 dark). Animals were housed individually, and four experimental groups were established: **NP-I** (*n* = 10), **AP-I** (*n* = 11), **NP-C** (*n* = 10), and **AP-C** (*n* = 9). Males were weighed weekly; glucose, testosterone, and insulin-like growth factor 1 (IGF-1) concentrations were monitored biweekly; and feed intake was measured every 3 wk from September to February. At 38 wk of age, males were slaughtered to assess carcass yield. Each animal was considered an experimental unit, and the data were analyzed using mixed models and repeated measures of SAS. Goats exposed to AP gained more weight and had greater final body weights than those under NP conditions (*P* < 0.001). Intact males also gained more weight than castrated males (*P* < 0.001). Feed intake was greater in AP-treated animals (*P* < 0.001). Carcass yield was greater in both AP- and I-males compared to NP- and C-males, respectively (*P* < 0.001). IGF-1 concentration increased in AP- and I-males than in NP- or C-males (*P* < 0.001), and glucose concentration increased in AP-treated animals compared to those exposed to NP (*P* < 0.001). Testosterone concentration increased in AP males than in NP males in weeks 15 and 17 (*P* < 0.01), but not at the other time points (*P* > 0.05). These findings suggest that exposure to artificial long-day photoperiods increases daily weight gain, feed intake, final body weight, and carcass yield in young male goats, regardless of their reproductive status. These improvements were associated with increased IGF-1 and glucose concentrations, while testosterone concentration remained unaffected.

## Teaser

Exposure to artificial long days increased weight gain, feed intake, and carcass yield, regardless of whether males are intact or castrated. Improvements were linked to elevated insulin-like growth factor 1 and glucose concentrations, highlighting the potential of photoperiod manipulation to optimize goat production.

## Introduction

Small ruminant production systems, including goats and sheep, play a vital role in global food security, particularly in semiarid and arid regions (Meza-Herrera et al., 2024; Navarrete-Molina et al., 2024). The growing global demand for animal-derived products, driven by rapid population growth, highlights the urgent need to improve production efficiency (Miassi and Dossa, 2023). Developing more productive and sustainable livestock systems is imperative, especially given the rising costs of animal feed, which are exacerbated during off-season periods. To address these challenges, implementing cost-effective and practical strategies to enhance livestock productivity while minimizing operational expenses is essential.

One promising strategy for improving productivity in small ruminants is the controlled use of artificial lighting. This low-cost intervention has been shown to influence reproductive and physiological processes in various species, leading to enhanced production outcomes (Chemineau et al., 1992; Dahl et al., 2000; Flores et al., 2011; 2018). In livestock raised in temperate regions, increasing the photoperiod from 8 to 16 h of light per day during winter has been reported to stimulate daily weight gain (**DWG**) compared to natural photoperiod (**NP**) conditions (Peters et al., 1978; Brinklow and Forbes, 1984; Suttie et al., 1991). Similarly, in gonad-intact lambs, exposure to an extended artificial photoperiod (**AP**) has been associated with greater growth rates compared to those maintained under shorter day lengths (Forbes et al., 1975). Although the mechanisms underlying this response remain incompletely understood, several factors have been proposed, including increased appetite and voluntary feed intake (Adam and Mercer, 2004), increased circulating concentration of insulin-like growth factor 1 (**IGF-1**; Jin et al., 2013; Pehlivan, 2019), and the anabolic effects of steroid hormones, particularly testosterone (Mahmood and Al-Obaidi, 2017). Furthermore, intact male lambs typically exhibit greater growth rates, feed efficiency, and carcass yield than castrated counterparts (Schanbacher et al., 1980). Interestingly, in intact lambs exposed to long-day photoperiods, reductions in circulating testosterone were observed alongside improvements in feed efficiency and final body weight, suggesting that photoperiod-induced growth responses may occur independently of gonadal function (Schanbacher and Crouse, 1980). Across species, these mechanisms (increased appetite and voluntary feed intake, increased circulating concentration of IGF-1, and the anabolic effects of steroid hormones) can be strategically targeted to promote the productive and behavioral traits. Accordingly, a deeper understanding and precise modulation of these endocrine and metabolic pathways may facilitate species-specific improvements in growth performance, reproductive efficiency, and feed conversion, while also addressing associated behavioral challenges.

In northern Mexico, young male goats exposed to a long-day AP (16 h light: 8 h dark) for 5 mo exhibited greater weight gain than those maintained under NP conditions (Vargas-Cruz et al., 2022). However, the physiological mechanisms underlying this response, whether due to changes in testosterone, IGF-1, or voluntary feed intake, remain unclear. To investigate this, we tested the hypothesis that the increase in body weight observed in young castrated male goats under APs is independent of circulating testosterone concentration. Castrated and intact males were exposed to an artificial long-day photoperiod for 35 wk, while control animals were maintained under the NP prevailing during the same season.

## Materials and Methods

### Ethical approval

All experimental procedures adhered to the *Technical Specifications for the Production, Care, and Use of Laboratory Animals* (NOM-062-ZOO-1999) of the Official Mexican Standards (SAGARPA, 2001). The Institutional Animal Care and Use Committee approved the protocol (Approval No. 13462034412).

### General study conditions

The study was conducted at INIFAP-CE La Laguna, located in Coahuila, Mexico (26°23′N, 104°47′W), from September to February. During this period, the average sunrise and sunset times were 7:24 a.m. and 6:36 p.m., respectively, resulting in an average NP of 11 h and 10 min. It is important to note that young male goats from both treatment groups were prenatally exposed to natural long-day photoperiods through their maternal melatonin signal (Deveson et al., 1992).

Forty young Creole male goats were selected from a commercial flock raised under extensive grazing conditions. All animals were born in June and were selected based on single birth type and similar birth age and weight. Until weaning, kids were nursed by their dams. From birth to weaning, young male goats were only exposed to NP. Starting from the third week of age, they were offered alfalfa and 100 g/day/head of commercial concentrate (Purina Calf Starter, 18% CP, Torreón, Coahuila, Mexico). At 5 wk of age (±0.1 wk), weaning occurred, and kids were permanently separated from their dams before being transported to INIFAP.

Castration was performed on nineteen young male kids 4 wk before the start of the experimental period, when the goats were approximately 8 wk old. The procedure followed the guidelines of the Farm Animal Welfare Committee (FAWC, 2008). Prior to surgery, sedation was achieved using xylazine (0.05 mg/kg body weight), and the scrotum was disinfected with iodine. A transverse incision was made at the distal end of the scrotum to remove the testicles. Hemostasis was achieved by ligating the spermatic cords using Dexon 2-0 EP3 absorbable polyglycolic acid sutures. The incision was completed below the ligature. Postoperative analgesia was administered via subcutaneous injection of meloxicam (1 mg/kg) to manage pain (Orihuela and Ungerfeld, 2019). From weaning to postcastration, young male goats were only exposed to NP.

Postcastration, males were housed individually and fed a basal diet consisting of 56% alfalfa hay, 12% soybean meal, and 30% rolled corn, formulated to provide 15.7% crude protein (**CP**) and 2.5 Mcal of metabolizable energy per kg of dry matter. The diet was supplemented with 1% sodium carbonate and 1% mineral mix (5% calcium, 75% sodium chloride, 17% phosphorus, 3% magnesium). Feed was offered ad libitum, twice daily at 8:00 a.m. and 2:00 p.m. The period from postcastration (week 8) to the initiation of light treatment (week 13) allowed the animals to adapt to their diet and individual pens.

### Experimental design

**Figure 1.**
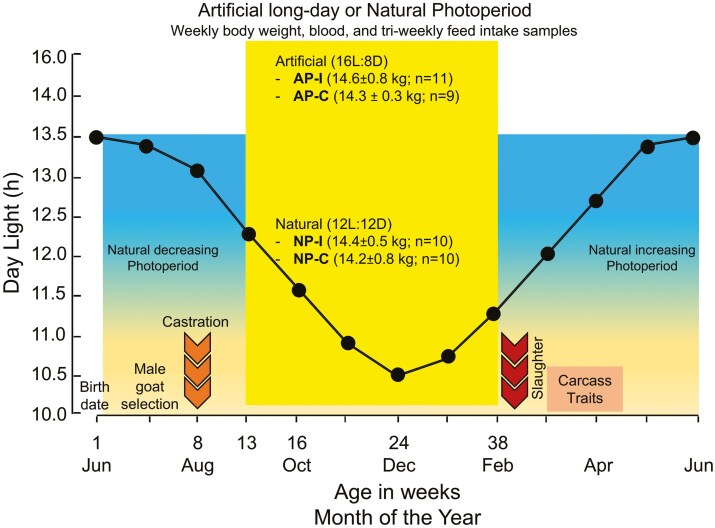
Schema of the experimental protocol. Forty young male goats born in June were selected from a commercial herd of 100 animals and castrated when they were 8 wk of age (left arrows). After a recovery period and at approximately 13 wk of age, castrated (C) and intact (I) males were subjected to AP (16 h light:8 h dark) or NP treatment (from September to February). Weekly body weights and blood samples were recorded until 9 mo of age. Beginning in week 17 and then every 3 wk thereafter, feed intake was measured until the end of the experiment. Males were slaughtered when they were 9 mo of age and carcass traits were recorded.

At approximately 13 wk of age, after the NP began decreasing in September, the goats were assigned to a completely randomized 2 × 2 factorial design (see Figure 1). The factors were: **Photoperiodic treatment**: Natural (NP) or Artificial (AP; 16 h light: 8 h dark), and **Reproductive condition**: Intact (I) or Castrated (C). This yielded four experimental groups, balanced by body weight: **NP-I**: Intact males under NP (14.4 ± 0.5 kg, *n* = 10). **AP-I**: Intact males under AP (14.6 ± 0.8 kg, *n* = 11). **NP-C**: Castrated males under NP (14.2 ± 0.8 kg, *n* = 10). **AP-C**: Castrated males under AP (14.3 ± 0.3 kg, *n* = 9).

### Photoperiod treatment

A 25 × 20 m pen was divided into two sections (20 × 2 m each). One section housed the NP groups in 20 individual pens (1 × 2 m), and the other housed the AP groups in similarly sized pens. The two sections were separated by 15 m. This pen allocation allowed the young male goats to have access to visual and auditory signals from other conspecifics, thereby avoiding social stress through isolation. To prevent light contamination, the AP pens were enclosed with blackout blinds. Artificial lighting (250-watt fluorescent lamps mounted 2 m above ground) delivered ~400 lux at eye level. Lights were controlled via a timer (TEM-08E, Steren, Mexico City) and operated from 6:00 to 9:00 a.m. and 5:00 to 10:00 p.m., extending the photoperiod to 16 h light daily. The AP treatment was maintained until the goats reached 35 wk of age.

### Measurements

#### Body weight

Goats were weighed weekly from week 11 until the end of the study (Figure 1), following a 12-h fast. Weighing was conducted in the morning using a portable scale (100 kg capacity, ± 0.05 kg). DWG was calculated as:


$$\begin{array}{l} DWG=(Finalweight-Initialweight)/Numberofexperimentaldays \end{array}$$

#### Feed intake

Beginning in week 17 and then every 3 wk thereafter, feed intake was measured. At 8:00 a.m. on the measurement day, feed troughs were emptied, and fresh feed was weighed and offered. Refusals were collected and weighed after 24 h. If all the feed was consumed, that amount was recorded as the intake.

#### Testosterone

Due to resource constraints, serum testosterone was measured biweekly in a randomly selected subset of intact males (*n* = 7 per treatment) from weeks 13 to 33 of age. Blood samples (5 mL) were obtained at 8:00 h by jugular venipuncture using tubes containing silica particles as a coagulation activator. Blood was allowed to clot for 1 h to obtain blood serum. Fibrin, blood cells, and platelets were separated from serum by centrifugation at 2,500 × *g* for 5 min and stored at −20 °C until analysis. Testosterone concentration was analyzed using a commercial ELISA kit (DRG Testosterone-EIA 1559, ALPCO, NJ, USA). The intra- and inter-assay coefficients of variation were 3.3% and 6.5%, respectively; assay sensitivity was 0.056 ng/mL. Testosterone was not assessed in castrated males.

#### Insulin-like growth factor 1

IGF-1 concentration was also assessed biweekly in a subset of males (*n* = 7 per treatment). The same intact goats used for testosterone were used; castrated goats were randomly selected. Blood samples were collected during fasting at 8:00 a.m. from weeks 13 to 33. Blood (5 mL) with the same protocol as for testosterone. Samples were centrifuged at 2,500 × *g* for 5 min, and serum was stored at −20 °C. IGF-1 was quantified using an ELISA kit (DRG IGF-1 600, ALPCO, NJ, USA). The intra- and inter-assay coefficients of variation were 3.39% and 6.55%, respectively; assay sensitivity was 0.02 ng/mL.

#### Glucose

Blood glucose concentration was measured biweekly from weeks 13 to 33 following a 12-h fast. Two drops of blood were collected by jugular venipuncture and immediately applied to a test strip for analysis using a handheld glucometer (Accu-Chek Sensor Comfort, Roche, Mexico). The glucometer had a detection range of 20 to 600 mg/dL.

#### Slaughter and carcass yield

At 38 wk of age, animals were fasted from feed and water for 12 h, then transported 20 km to a commercial slaughterhouse in Matamoros, Coahuila, Mexico. Slaughter procedures followed the Official Mexican Standard for the humane killing of domestic and wild animals (NOM-033-SAG/ZOO-2014). Goats were randomly selected for slaughter and handled in accordance with standard ante-mortem and postmortem protocols to ensure their welfare and carcass quality. Preslaughter body weight was recorded immediately before slaughter. Carcass weight was determined after removal of skin, viscera, head, and feet using a portable scale.

#### Statistical analysis

Data analyses were conducted using the SAS statistical package SAS version 9.3 (2010). Each animal was considered an experimental unit. The data were analyzed with Shapiro–Wilks test to verify the normal distribution (PROC-UNIVARIATE). Body weight, glucose, testosterone, IGF-1, and feed intake were analyzed using PROC-MIXED (SAS, 2010). Fixed effects in these analyses were photoperiod (natural and artificial) and reproductive condition (intact and castrated). For these previous variables, the different sampling dates were included as repeated measures. Post hoc analyses among means for treatments for variables measured at different time points during the experiment were analyzed using LSD of PROC GLM. Data were presented as mean ± SEM. A *P*-value of ≤0.05 was considered significant.

## Results

### Body weight and DWG

**Figure 2.**
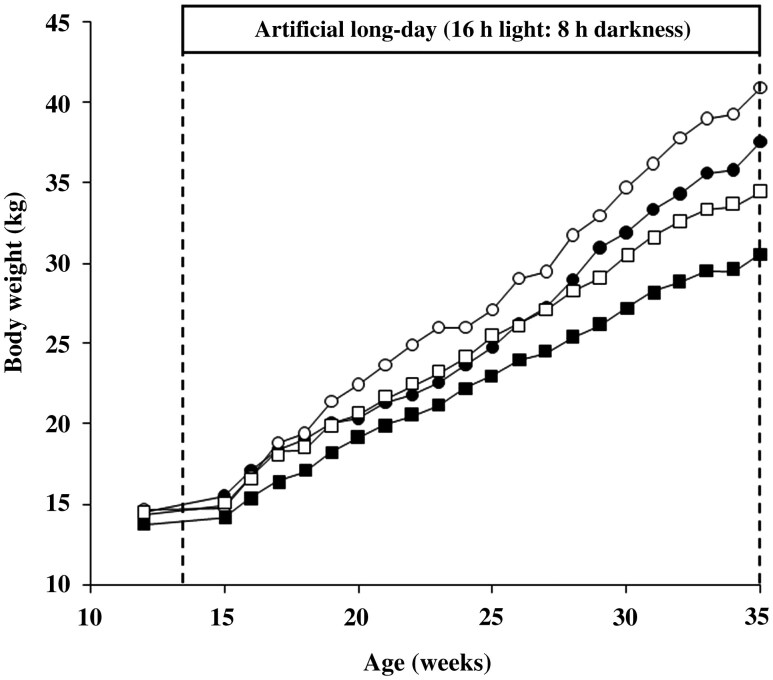
Average body weight of intact young male goats maintained in NP (●: *n* = 10) or exposed to an AP (○: *n* = 11). Two other groups of castrated males were maintained in an NP (■: *n* = 10) or exposed to an AP (□: *n* = 9).

Male goats exposed to the AP gained more weight than those maintained under the NP, regardless of reproductive condition (Figure 2; *P* < 0.05). Intact males gained more weight than castrated males (Figure 2; *P* < 0.001). No significant interaction was observed between photoperiodic treatment and reproductive condition on body weight (*P* > 0.05). As expected, body weight increased with age (*P* < 0.001). DWG was also greater in AP males (145 g/d) compared to NP males (123 g/d; *P* < 0.001). Similarly, intact males had a greater DWG (153 g/d) than castrated males (114 g/d; *P* < 0.001). The interaction between photoperiod and reproductive condition was not significant for DWG.

### Feed intake

Feed intake was greater in AP males than in NP males (Table 1; *P* < 0.01) and in intact males compared to castrated males (Table 1; *P* < 0.001). No significant interaction was found between photoperiodic treatment and reproductive condition on feed intake. Feed intake increased with age (*P* < 0.001), and this increase was more pronounced in AP males than in NP males (*P* < 0.001).

**Table 1.** Average (±SEM) feed intake (g/d) of young intact (NP-I) or castrated males (NP-C) maintained under a NP or exposed to an AP-I [intact or AP-C [castrated])

	Groups (g/d)					*P*-value		
Age (weeks)	NP-I	NP-C	AP-I	AP-C	SEM	PF	C	PF*C
17	662^ab^	582^a^	739^b^	628^ab^	176	0.01	0.001	>0.05
20	953^ab^	942^a^	1082^b^	1024^ab^	251			
23	1101^b^	1154^ab^	1181^b^	1334^c^	140			
26	1162^ab^	1064^a^	1306^b^	1182^ab^	217			
29	1104^ab^	953^b^	1232^a^	1090^ab^	278			
32	1362^a^	1202^bc^	1320^ac^	1138^b^	207			
35	1220^bc^	994^b^	1315^a^	1252^c^	315			

Abbreviations: PF, photoperiod effect; C, reproductive status (intact or castrated); PF*C, interaction. Different superscripts indicate significant differences between treatments.

### Carcass yield

Slaughter weight was greater in AP males (37.9 ± 1.1 kg) compared to NP males (34.0 ± 1.3 kg; *P* < 0.01), and in intact males (39.3 ± 1.2 kg) compared to castrated males (32.4 ± 0.8 kg; *P* < 0.001). Similarly, carcass weight was greater in AP males (19.0 ± 1.2 kg) than in NP males (17.0 ± 1.4 kg; *P* < 0.01), and in intact males (19.3 ± 0.64 kg) compared to castrated males (16.5 ± 0.5 kg; *P* < 0.001).

Carcass yield was greater in AP males (50.5%) compared to NP males (48.5%; *P* < 0.001). However, the reproductive condition did not affect carcass yield (intact: 49.7% vs castrated: 48.8%; *P* > 0.05). There were no significant interactions between photoperiodic treatment and reproductive condition on slaughter weight, carcass weight, or carcass yield (*P* > 0.05).

### Testosterone

**Figure 3.**
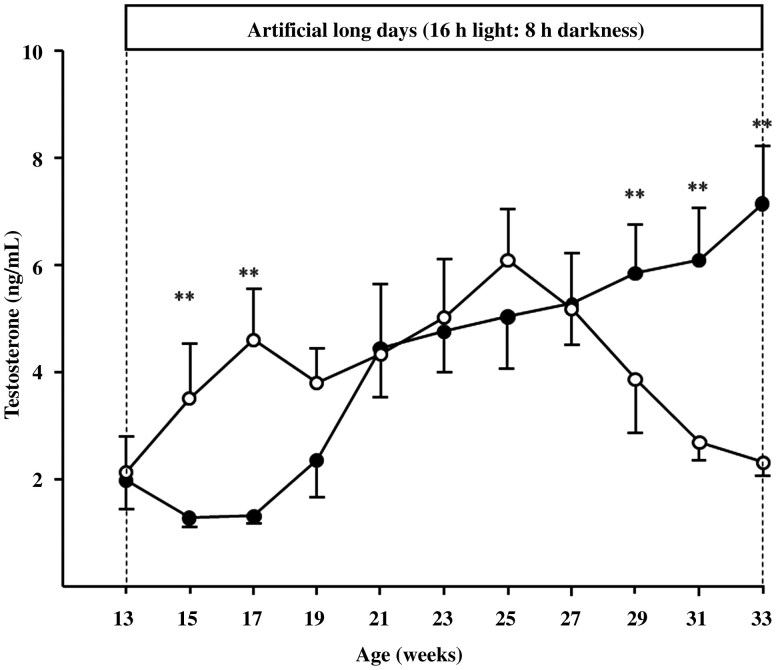
Mean (±SEM) testosterone concentrations of young male goats maintained in NP (●: *n* = 10) or exposed to an artificial long-day photoperiod (○: *n* = 11). ** = significant differences (*P* < 0.01) between experimental groups.

Figure 3 shows plasma testosterone concentration in intact males exposed to either artificial or NPs. At the beginning of the experiment, testosterone concentration did not differ between AP (2.0 ± 0.3 ng/mL) and NP males (2.1 ± 0.9 ng/mL; *P* > 0.05). However, at 2 and 4 wk after the onset of artificial long-day exposure, testosterone concentration increased in AP males than in NP males (Figure 3; *P* < 0.01). Subsequently, testosterone concentration converged between groups (*P* > 0.05). However, toward the end of the experiment, NP males exhibited increased testosterone concentration compared to AP males (Figure 3; *P* < 0.01).

### Insulin-like growth factor 1

**Figure 4.**
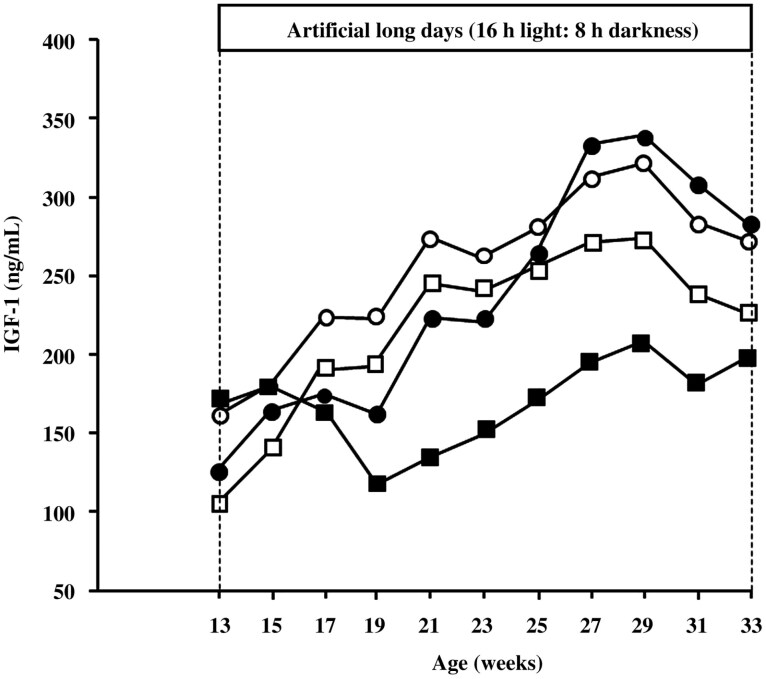
IGF-1 concentrations of intact young male goats maintained in NP (●: *n* = 10) or exposed to an AP (○: *n* = 11). Two other groups of castrated males were maintained in an NP (■: *n* = 10) or exposed to an AP (□: *n* = 9).

IGF-1 concentration increased in AP males compared to NP males, independent of reproductive condition (Figure 4; *P* < 0.001). Intact males also had increased IGF-1 concentration than castrated males (Figure 4; *P* < 0.001). A significant interaction was observed between photoperiodic treatment and reproductive condition on IGF-1 concentration (*P* < 0.05).

### Glucose

Glucose concentration increased in AP males compared to NP males (*P* < 0.001; Table 2). No significant differences in glucose concentration were observed between intact and castrated males (*P* > 0.05). The interaction between photoperiodic treatment and reproductive condition on glucose concentration was not significant (*P* > 0.05).

**Table 2.** Mean glucose concentration (± SEM) of intact young male goats maintained under NP-I or exposed to an AP-I

	Groups (mg/dL)					*P*-value		
Age (weeks)	NP-I	NP-C	AP-I	AP-C	SEM	PF	C	PF*C
13	68	70	68	68	8	0.001	> 0.05	> 0.05
15	68	68	73	70	10			
17	69^a^	70^a^	79^b^	83^b^	14			
19	73^a^	72^a^	78^b^	76^b^	13			
21	67^a^	68^a^	75^ab^	76^b^	10			
23	72^a^	72^a^	77^a^	77^a^	11			
25	73^ab^	69^b^	78^a^	75^ab^	10			
27	81^a^	84^a^	80^a^	83^a^	14			
29	75^a^	73^a^	78^b^	76^a^	8			
31	85^a^	83^a^	83^a^	77^a^	16			
33	68^ab^	64^a^	70^ab^	74^b^	11			

Abbreviations: PF, photoperiod effect; C, reproductive status (intact or castrated); PF*C, interaction. Different superscript letters indicate statistical significance (*P* < 0.05).

Two other groups of castrated males were kept under NP-C, while the other group was subjected to artificial long days (AP-C).

## Discussion

This study investigated the effects of an artificial long-day photoperiod (AP; 16 h light: 8 h dark) on growth performance and metabolic parameters in young male goats, compared to animals maintained under a naturally decreasing photoperiod (NP). Male goats exposed to AP exhibited increased feed intake and body weight gain relative to NP counterparts, regardless of reproductive status (intact or castrated). Furthermore, intact males gained more weight than castrated males, independent of photoperiodic treatment. Notably, AP exposure led to improved carcass weight and yield.

The observed changes in body weight were aligned with increased concentrations of IGF-1 and glucose, but not testosterone, in animals exposed to the AP. DWG, final body weight, and blood glucose concentration increased in AP-exposed males compared to those under the NP, in agreement with the findings of Vargas-Cruz et al. (2022). While their study suggested a potential role for testosterone in promoting weight gain due to its well-established anabolic effects (Schanbacher et al., 1980), our results provide further clarification. In our study, males exposed to AP showed greater weight gain, although there was no overall difference in testosterone concentration between the AP and NP groups. We acknowledge the anabolic properties of testosterone on growth performance, but its concentration was increased only at two specific sampling points in the AP group. These findings support the hypothesis that the photoperiod itself, rather than testosterone, is the primary factor driving the observed increase in body weight.

The more pronounced weight gain observed in intact AP-exposed males compared to castrated counterparts further supports the hypothesis of a direct photoperiodic effect on growth. This finding is consistent with the results of Schanbacher and Crouse (1980), who reported greater weight gain in intact versus castrated Suffolk lambs under a long-day photoperiod, despite lower testosterone concentration. In the present study, the secretory profile of testosterone exhibited fluctuations over time, as previously described by Ahmad et al. (1996) and Rosales-Nieto et al. (2024). Although testosterone concentration increased shortly after AP exposure, no significant differences were observed between AP and NP groups throughout the experimental period. In NP males, testosterone concentration increased gradually, reaching a peak at 33 wk of age. The absence of circulating androgens in castrated males likely contributed to reduced muscle mass synthesis, lower glycogen storage capacity, and increased visceral fat deposition (Bardin, 1996; Anukulkitch et al., 2007), thereby accounting for their lower weight gain.

The enhanced growth observed in AP-exposed males may also be attributed to increased IGF-1 concentration. IGF-1, regulated by growth hormone, plays a critical role in promoting cellular growth, differentiation, and function, as well as in inhibiting apoptosis across various tissues in domestic animals (Akers, 2006). Similar observations have been made in lactating females and goat kids (Flores et al., 2015; 2018), with long-day photoperiods shown to enhance IGF-1 via growth hormone stimulation (Jin et al., 2013). Therefore, the increased IGF-1 in AP-exposed males likely contributed to their rapid weight gain and increased feed intake compared to NP controls, consistent with prior studies (Suttie et al., 1991; Francis et al., 1997; Webster et al., 2001).

The increased blood glucose concentration in AP males further suggests an anabolic metabolic state (Archer et al., 2004), which may have also contributed to the enhanced weight gain. This increase in glucose likely reflects greater feed intake, as AP animals consumed 9% more feed than NP animals. This observation is consistent with previous reports of increased feed intake (7% to 12%) in heifers and lambs exposed to long photoperiods (Peters et al., 1980; Schanbacher and Crouse, 1980). Mechanisms underlying increased intake under long-day conditions may include both behavioral factors (e.g., meal size and frequency; Rhind et al., 2002) and endocrine modulation involving hormones such as prolactin, IGF-1, thyroxine (T4), triiodothyronine (T3), and leptin (Rhind and McMillen, 1995; Bocquier et al., 1998; Rhind et al., 1998; Marie et al., 2001). These hormones likely interact with peripheral organs and the gut to promote body growth.

AP treatment not only enhanced overall body weight but also improved carcass weight and yield. For example, lambs fed ad libitum or on restricted diets under long APs exhibited greater carcass weights (26.6 kg) than those under short-day conditions (24.8 kg; Forbes et al., 1975). Consistent with this, our study found greater carcass yield in AP-exposed goats, supporting earlier reports that long-day photoperiods increase carcass weight and fat deposition in sheep and lambs (Vernon et al., 1986; Bocquier et al., 1998; Thiéry et al., 2002). These effects are thought to result from increased metabolic activity in adipose and muscle tissue, potentially mediated by enzymes such as lipoprotein lipase (Bocquier et al., 1998; Faulconnier et al., 2001; Klein-Júnior et al., 2006). Although we did not assess carcass fatness in this study, a limitation, the findings suggest that long-day photoperiods may enhance fat deposition and improve growth efficiency. This has potential practical relevance for optimizing goat production during periods of seasonal reproductive quiescence (Yu et al., 2021).

### Strengths and limitations

A major strength of this study is the demonstration that artificial long-day photoperiods improve key performance metrics, including weight gain, feed intake, final body weight, and carcass yield, in young male goats, regardless of reproductive status. These improvements were accompanied by increased IGF-1 and glucose concentrations, with no corresponding changes in testosterone concentration, underscoring the independent effect of photoperiod. The applicability of these findings across various goat production systems enhances their significance. However, a limitation of this study is the lack of carcass fatness evaluation, which could have further clarified the contribution of fat deposition to weight gain. Additionally, resource constraints limited both the sample size per treatment group and the number of hormone measurements (IGF-1 and testosterone). Nonetheless, the consistency of the observed outcomes supports the potential of artificial lighting as a practical and sustainable strategy to enhance production efficiency in male goats.

## Conclusion

In summary, artificial long-day photoperiod exposure in young male goats increases feed intake, body weight gain, and carcass yield. These effects were aligned with increased IGF-1 and glucose concentrations, suggesting a direct role of photoperiod in growth enhancement. The secretory profile of testosterone exhibited fluctuations over time in both groups, and testosterone concentration increased in AP males at specific time points. These findings offer valuable insights into the potential of photoperiod manipulation as a practical approach for enhancing growth performance in male goats.

## Data Availability

None of the data was deposited in an official repository. The data supporting this study’s findings are available from the corresponding author upon reasonable request.

## Abbreviations

CP: crude protein
DM: dry matter
DWG: daily weight gain
IGF-1: insulin growth factor 1
ME: metabolizable energy
